# Enhanced SAR Compression through Multi-Look Doppler Compensation and Auto-Focusing Technique

**DOI:** 10.3390/s24206551

**Published:** 2024-10-11

**Authors:** Hyeon Seong Kim, Yong Hwi Kwon, Chul Ki Kim

**Affiliations:** The School of Electronic Engineering, Soongsil University, Seoul 06978, Republic of Korea; confide124@soongsil.ac.kr (H.S.K.); rnjsdydgnl2@soongsil.ac.kr (Y.H.K.)

**Keywords:** synthetic aperture radar, doppler estimation, auto-focusing, fractional Fourier transform

## Abstract

This paper presents a simple and streamlined compensation technique for improving the quality of synthetic aperture radar (SAR) images based on the Range Doppler Algorithm (RDA). Incorrect Doppler estimation in the space orbit, caused by unexpected radar motion errors, orbit mismatches, and other factors, can significantly degrade SAR image quality. These inaccuracies result in mismatches between the azimuth-matched filter and the received Doppler chirp signal. To address this issue, we propose a Doppler estimation method that leverages the Fractional Fourier Transform (FrFT) and cross-correlation techniques. The received signals are compared with the azimuth-matched filter based on the rotation angle in the FrFT domain, and the Doppler centroid is adjusted to achieve the optimal alignment. This process ensures high correlation values and enhanced resolution in the final SAR image. The efficacy of the proposed technique is validated through experiments using real spaceborne SAR data from the practical satellite. The results demonstrate significant improvements in image quality and resolution compared to conventional algorithms, highlighting the advantages of our approach for various remote sensing applications.

## 1. Introduction

Synthetic aperture radar (SAR) is an advanced remote sensing technology used to monitor and analyze the Earth’s surface through radar systems mounted on platforms such as satellites and aircraft [1,2,3]. Initially developed for military purposes, SAR technology was first operationalized with the launch of the SEASAT satellite in 1978. Today, synthetic aperture radar is widely employed in civilian applications, such as terrain mapping of inaccessible regions. One key advantage of SAR is the ability to synthesize a much larger antenna aperture, resulting in higher-resolution images than the physical antenna length would allow. Additionally, synthetic aperture radar operates effectively in all weather conditions due to the properties of the electromagnetic waves utilized. This capability to produce high-resolution images under various conditions has driven significant research in target analysis and method development. Recently, the SAR techniques integrated within machine learning, video processing, and other achievements have been actively emerging in many radar research fields and are receiving a lot of attention in a wide range of applications [4,5]. The Range Doppler Algorithm (RDA) is one of the foundational algorithms for signal processing techniques, initially developed for civilian satellite SAR operations [6]. The widespread use of RDA is due to its maturity, simplicity, efficiency, and accuracy [7]. In synthetic aperture radar signal processing with RDA, there are two main signals: the received signal, which is captured by the radar system for target information analysis, and the matched filter signal, which is generated to process the received signal [8]. Conventional techniques assume that the azimuth-matched filter is estimated perfectly as the azimuth signal in the received data, resulting in an optimal synthetic aperture radar image. However, the RDA also has limitations, particularly in scenarios with inaccurate Range Cell Migration Correction (RCMC) and the integration of azimuth frequency for secondary range compression [9]. To address these issues, various alternative algorithms have been proposed [10,11]. Moreover, in practical implementations, SAR images often degrade due to not properly estimating the azimuth-matched filter. The above issues of RDA can be caused mainly by unexpected Doppler estimation errors such as variations in radar platform velocity, terrain distortions, solar wind effects, etc. These Doppler estimation inaccuracies can significantly impair SAR image quality, leading researchers to explore various calibration methods to mitigate these issues. Although the existing literature offers numerous techniques for enhancing SAR image quality [12,13], these methods are often complex, non-intuitive, and should be modified according to specific situations, making them difficult to apply in all environmental conditions. Some methods are designed to operate only in specific cases making them restrictive technologies [14,15,16]. The reference techniques propose the advanced Doppler centroid estimator and the results of these show high SNR in the specific situation and deal with several data for learning and training. It still needs more processes, high complexity, and increased processing time.

This paper introduces an efficient and streamlined compensation algorithm that is intuitive and straightforward, focusing on improving synthetic aperture radar image quality. The proposed technique is particularly well-suited for CubeSats, which require miniaturized, optimized, and cost-effective solutions. The simplicity and efficiency of our method ensure its high versatility and ease of implementation on these compact platforms. The algorithm can be applied to various situations in satellite platforms and handle general Doppler errors, making it simpler and easier to understand without the need to derive new equations. The proposed method emphasizes correcting two critical aspects: the Doppler chirp rate and the Doppler centroid frequency. The primary issue addressed is phase distortion of the received signal, which adversely impacts synthetic aperture radar image quality through improper matched filtering. The key contribution of this paper is compensating for Doppler effects using the tools of the Fractional Fourier Transform (FrFT) and cross-correlation techniques. During the range compression stage of RDA, raw data are efficiently processed using a range-matched filter. However, accurate Doppler estimation becomes crucial during azimuth compression to prevent the degradation of matched filtering. Discrepancies in chirp rates between the two signals result in residual phase errors, leading to suboptimal azimuth-matched filtering. The Fractional Fourier Transform technique provides insight into signal rates by rotating the time–frequency domain, allowing for iterative adjustments of the azimuth-matched filter’s chirp rate to align with the received signal. This adjustment ensures the azimuth-matched filter has the same Doppler chirp rate as the raw signals in the azimuth direction. An incorrect Doppler centroid estimation further complicates azimuth-matched filtering due to the resultant phase errors. The error of the Doppler signal estimation is usually caused by such factors as the velocity variance of platforms, noise/interference environment, irregularity of terrain, etc. Cross-correlation techniques can correct for unexpected errors. An inaccurate Doppler centroid estimation implies misaligned Doppler centroids in the time–frequency domain, reducing the correlation value and degrading azimuth compression results. By iteratively adjusting the Doppler centroid of the azimuth-matched filter to maximize correlation, proper signal alignment is ensured. The combined application of Fractional Fourier Transform and cross-correlation accurately estimates the chirp rate and Doppler centroid of the raw signal, thus enhancing the final SAR image quality.

This paper is structured as follows: Section 2 provides a theoretical background on the Range Doppler Algorithm, cross-correlation, and Fractional Fourier Transform. Section 3 details the proposed algorithm for optimizing synthetic aperture radar images. Section 4 presents the experimental results using real synthetic aperture radar data and compares them with the results obtained using the original algorithm. Finally, Section 5 concludes the paper.

## 2. Background Theory

### 2.1. Range Doppler Algorithm

**Figure 1:**
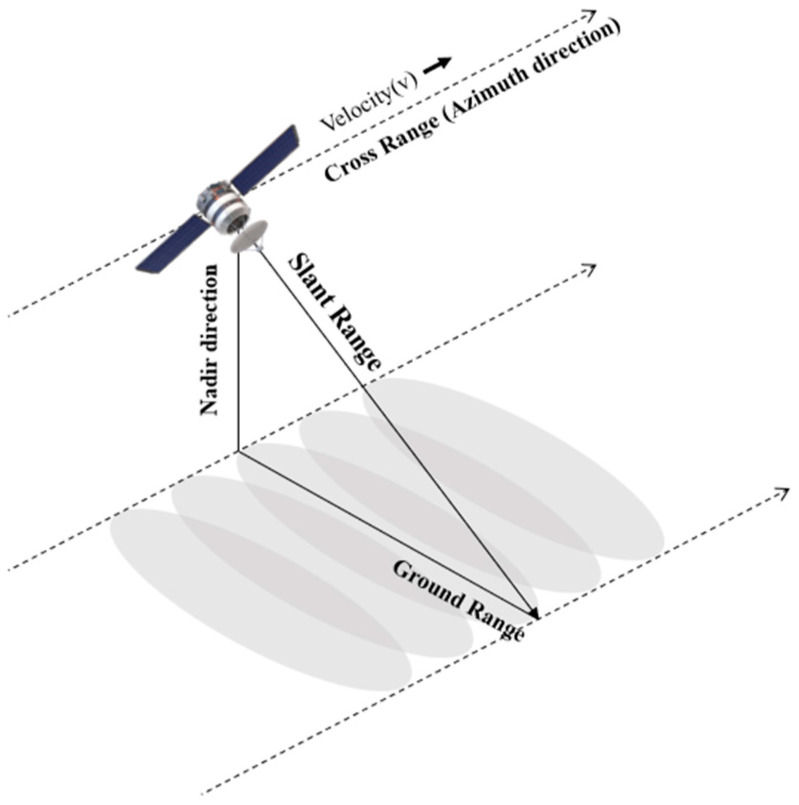
The strip-mode operation in spaceborne-SAR.

**Figure 2:**
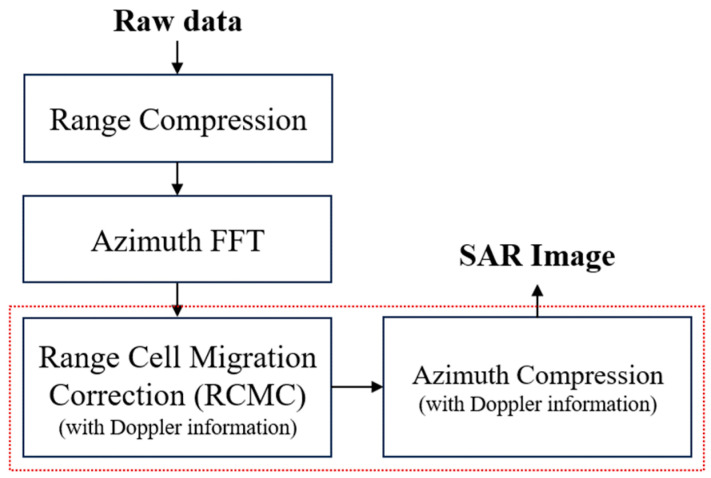
Overview of the Range Doppler Algorithm.

The Range Doppler Algorithm (RDA) is a prominent method for extracting synthetic aperture radar (SAR) images [12]. Among the various SAR operating modes, strip-mode is commonly employed in conjunction with the RDA in SAR systems. An illustration of strip-mode is provided in Figure 1. The RDA processes signals in both the range direction (direction of propagation) and the azimuth direction (direction of radar movement). This method is considered straightforward and user-friendly compared to other SAR processing techniques such as the Chirp Scaling Algorithm and the Omega-K algorithm. The conventional RDA is divided into three primary components: range compression, Range Cell Migration Correction (RCMC), and azimuth compression. The overall procedure of the RDA is depicted in Figure 2. In the context of SAR systems, range compression involves compressing the received echo signal to improve resolution in the range direction. The RCMC step corrects the migration of range cells caused by the relative motion between the radar platform and the target. Finally, azimuth compression enhances the resolution in the azimuth direction by focusing the signal along the radar’s flight path.

Definitions of raw data can vary depending on the operational characteristics of the radar systems, including FMCW (Frequency Modulated Continuous Wave), chirp pulse, and de-ramping techniques. Furthermore, SAR measurements are conducted across various measurement platforms (such as strip-map mode, spotlight mode, scan mode, etc.), resulting in differences in raw data characteristics. For the purposes of this paper, the raw data are specifically defined for the chirp pulse radar and strip-map mode to introduce the RDA comprehensively. To introduce RDA in this paper, we specifically define the raw data for the chirp pulse radar:

(1) $$S_{o}\left(\tau ,\eta \right)=A_{0}\omega_{r}\left[\tau -\frac{2R\left(\eta \right)}{c}\right]\omega_{a}\left(\eta -\eta_{c}\right)e^{-j4\pi f_{0}R(\eta )/c}e^{j\pi K_{r}{\left(\tau -2R(\eta )/c\right)}^{2}}$$

where $A_{0}$ is an amplitude of signal, $f_{0}$ is a radar center frequency, τ and η are the range and azimuth time, respectively, R(η) is the instantaneous slant range, $K_{r}$ is the range chirp FM rate, $\omega_{r}\left(\tau \right)$ is the range envelope, and $\omega_{a}\left(\eta \right)$ is the azimuth envelope. For target compression in the range direction, the matched filter is defined as:

(2) $$G\left(f_{\tau }\right)=rect(\frac{f}{KT})e^{-j\pi f^{2}/K}$$

The resulting range-compressed signal is defined as:

(3) $$S_{rc}\left(\tau ,\eta \right)=A_{0}p_{r}\left[\tau -2R(\eta )/c\right]\omega_{a}\left(\eta -\eta_{c}\right)e^{-j4\pi f_{0}R(\eta )/c}$$

where $p_{r}(\tau )$ is the IFFT of the $W_{r}\left(f_{\tau }\right)$, the range envelope in the range frequency domain. The measured location of each target signal can be curved due to differences in the instantaneous slant range along the azimuth direction. Therefore, the RCMC process rearranges the azimuth data of the same target, interpolating the range location by $R_{0}$, where $R_{0}$ is a zero Doppler distance. It means the nearest range between the platform and the target. For the target range from the radar, the range equation can be approximated as:

(4) $$R\left(\eta \right)=\sqrt{R_{0}^{2}+V_{r}^{2}\eta^{2}}\approx \;R_{0}+\frac{V_{r}^{2}\eta^{2}}{2R_{0}}$$

Furthermore, since Doppler signals are processed in the region where Doppler changes linearly, the FM rate can be defined as:

(5) $$K_{a}\approx \;\frac{2V_{r}^{2}}{{\lambda R}_{0}}$$

Based on (4) and (5), the range-compressed signal in the azimuth FFT domain can be shown as:

(6) $$S_{1}\left(\tau ,f_{\eta }\right)\approx \;A_{0}p_{r}\left[\tau -\frac{2R_{rd}\left(f_{\eta }\right)}{c}\right]\omega_{a}\left(f_{\eta }-f_{\eta_{c}}\right)e^{-j4\pi f_{0}R_{0}/c}e^{j\pi \frac{f_{\eta }^{2}}{K_{a}}}$$

where $f_{\eta }$ and $f_{\eta_{c}}$ represent the azimuth frequency and the Doppler centroid frequency, respectively, and the Range Cell Migration (RCM) in the range envelope, $R_{rd}\left(f_{\eta }\right)$, can be expressed in the range Doppler domain as shown below:

(7) $$R_{rd}\left(f_{\eta }\right)=R_{0}+\frac{\lambda^{2}R_{0}f_{\eta }^{2}}{8V_{r}^{2}}$$

We can regard this signal as a function of range variant, $R_{0}$. The RCMC is performed by the interpolation in the range Doppler domain and the amount of RCM is relocated at $R_{0}$.

(8) $$S_{2}\left(\tau ,f_{\eta }\right)\approx \;A_{0}p_{r}\left[\tau -\frac{2R_{0}}{c}\right]\omega_{a}\left(f_{\eta }-f_{\eta_{c}}\right)e^{-j\pi f_{0}R_{0}/c}e^{j\pi \frac{f_{\eta }^{2}}{K_{a}}}$$

We can confirm that the signal expressed in terms of $R_{0}$ and $p_{r}$ is independent of the range envelope. After the RCMC, the matched filtering is applied to the data, $S_{2}\left(\tau ,\eta \right)$, for the azimuth compression process. The azimuth-matched filter is estimated as shown below:

(9) $$H_{az}\left(f_{\eta }\right)=e^{-j\pi \frac{f_{\eta }^{2}}{K_{a}}}$$

To estimate an accurate azimuth-matched filter, the Doppler information (centroid frequency and frequency rate) is an important key parameter. Various environmental conditions, such as the velocity of SAR measurement, terrain distortion, solar wind, etc., can generate this Doppler effect. We suggest the estimation method for the above Doppler effect in the proposed technique. After the azimuth compression, the final signal in the frequency and time domains can be defined, respectively, as:

$$S_{ac}\left(\tau ,f_{\eta }\right)=A_{0}p_{r}\left[\tau -\frac{2R_{0}}{c}\right]\omega_{a}\left(f_{\eta }-f_{\eta_{c}}\right)e^{-j\frac{4\pi f_{0}R_{0}}{c}}$$

(10) $$s_{ac}\left(\tau ,\eta \right)=A_{0}p_{r}\left[\tau -\frac{2R_{0}}{c}\right]p_{a}\left(\eta \right)e^{-j\frac{4\pi f_{0}R_{0}}{c}}e^{j2\pi f_{\eta_{c}}\eta }$$

where $p_{a}$ and $p_{r}$ are sinc-like functions. As a result, we can notice that the final target is positioned at $\tau =2R_{0}/c$ and η = 0 in the range and azimuth direction, respectively.

### 2.2. Cross-Correlation Technique

**Figure 3:**
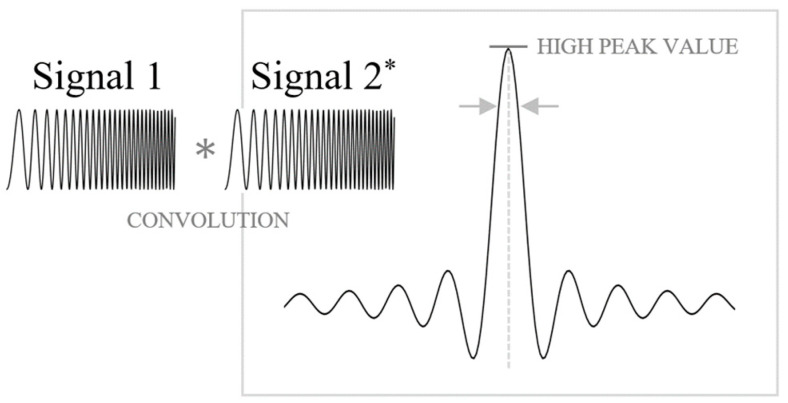
Cross-correlation processing between two chirp pulses (‘signal 1’ is the original signal and ‘signal 2*’ is the conjugated signal of signal 1) and the process result (relativity).

The cross-correlation method is a signal-processing technique used to measure and define the relationship between two signals [17] using the concept of convolution. As shown in Figure 3, the cross-correlation process evaluates the similarity between two chirp pulses. The higher the similarity between the two signals, the higher the expected peak value of the sinc function. Conversely, a lower similarity results in a lower peak value. Consider two identical chirp pulse functions, $f\left(\tau \right)$ and $g\left(\tau \right)$, which undergo cross-correlation. $f_{01}$ and $f_{02}$ denote the center frequencies of $f\left(\tau \right)$ and $g\left(\tau \right)$, respectively. The cross-correlation is defined as:

(11) $$\left(f\;*\;g\right)\left(\tau \right)=\int_{-\infty }^{\infty }f(t)g\left(t+\tau \right)\mathrm{d}t$$

where $g\left(t+\tau \right)$ represents the time-shifted version of $g\left(t\right)$. By computing the sliding dot product of $f\left(\tau \right)$ and $g\left(\tau \right)$, the cross-correlation value can be estimated. The primary comparative information determining the cross-correlation result is the center frequency and chirp rate of the chirp signals. The cross-correlation result, $\left(f\;*\;g\right)\left(\tau \right)$, is presented as a sinc function. Due to the linearly increasing frequency of the chirp pulse, the peak point appears when the two functions overlap perfectly. Therefore, the intersection point between $f_{01}$ and $f_{02}$ represents the maximum value of the sinc function, which is a characteristic property of the chirp pulse in cross-correlation. In the context of the Range Doppler Algorithm (RDA), cross-correlation operates in two dimensions: range and azimuth. The application of cross-correlation to the RDA is defined as follows [18]:

(12) $$\left(s_{Look1}\;*\;s_{Look2}\right)\left(\tau_{n},\;\eta \right)≝\;\int_{-\infty }^{\infty }s_{Look1}\left(\tau_{n}\right)s_{Look2}\left(\tau_{n},\eta +\omega \right)d\omega$$

If the chirp pulse signals exhibit high similarity, the cross-correlation value approximates the ideal sinc function in the frequency domain. This property is particularly useful for azimuth compression. In azimuth compression, the relative relationship between two separate signals—the received signal and the azimuth-matched filter—can be computed using a cross-correlation process. According to the similarity between Equations (8) and (9), the cross-correlation value in azimuth compression can be increased, enhancing the SAR image quality. By leveraging the properties of cross-correlation, specifically the alignment and similarity of chirp signals, the proposed method improves the precision of azimuth compression in SAR processing. It leads to higher quality and more accurate SAR images, which are crucial for various applications in remote sensing and terrain analysis.

### 2.3. Fractional Fourier Transform

**Figure 4:**
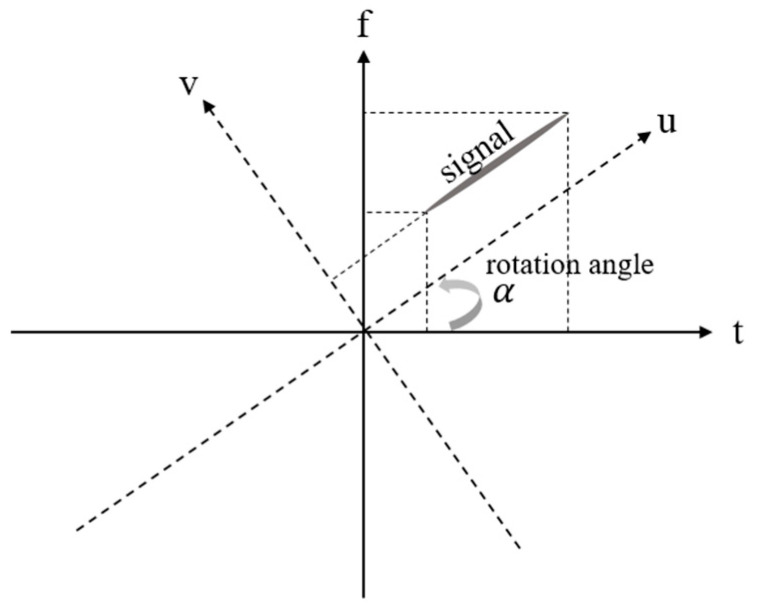
The time–frequency domain (t, f) and FrFT domain (u, v), which is the time–frequency domain rotated by the rotation angle, α.

The Fractional Fourier Transform (FrFT) is a generalization of the classical Fourier transform used in time–frequency representations [19]. Figure 4 illustrates a signal $x\left(t\right)$ in both the original time–frequency domain (solid line) and the rotated time–frequency domain (dashed line). As shown in Figure 4, the transformed signal in the FrFT domain exhibits varying degrees of concentration depending on the rotation angle. This variation is evidenced by the bandwidth of the signal extending across the frequency domain. The parameter “α” represents the rotation angle of the time–frequency domain. Notably, the Fourier transform is a special case of the FrFT, corresponding to a counterclockwise rotation by α =π/2. The FrFT can be mathematically defined as:

(13) $$X_{\alpha }\left(u\right)={FrFT}_{\alpha }\left(x\left(t\right)\right)=\int_{-\infty }^{\infty }x\left(t\right)K_{\alpha }\left(t,u\right)\mathrm{d}t$$

The transformation kernel, $K_{\alpha }\left(t,u\right)$, is defined as

(14) $$K_{\alpha }\left(t,u\right)=\left\{\begin{array}{c} \sqrt{\frac{1-jcot\alpha }{2\pi }}e^{j\frac{{(u}^{2}{+t}^{2})}{2}cot\alpha -jutcsc\alpha }\;if\;\alpha \neq n\pi \\ \delta \left(t-u\right)\;\;\;\;\;\;\;\;\;\;\;\;\;\;\;\;\;\;\;\;\;\;\;\;\;\;\;if\;\alpha =2n\pi \\ \delta \left(t+u\right)\;\;\;\;\;\;\;\;\;\;\;\;\;\;\;\;if\;\alpha =(2n+1)\pi \end{array}\right.$$

where δ(t) represents the Dirac delta function [20]. The inverse of the FrFT can be obtained by applying the FrFT with a rotation of −α:

(15) $$x(t)={FrFT}_{-\alpha }\left(X_{\alpha }\left(u\right)\right)=\int_{-\infty }^{\infty }X_{\alpha }\left(u\right)K_{-\alpha }\left(u,t\right)\mathrm{d}t$$

By the process of calculating (15), we can find the maximum integrated value when the transformation kernel is matched with the received signal. It means that the received signal is rotated by the kernel at an angle α. Thus, the chirp rate of the received signal can be parallel to the α-domain, as shown in Figure 4. Using the above features, we find the kernel that best suits the received signal and extract the highest integral value. More details of how to use the Fractional Fourier Transform for compensating Doppler estimation are introduced in the next section. In addition, there are several properties of the Fractional Fourier Transform that facilitate signal processing [21]. For example, the linearity, time-shifting, and modulation properties of the FrFT are similar to those of the classical Fourier transform, but they operate within the context of fractional domains, providing enhanced flexibility for analyzing and manipulating signals. In the proposed technique, we use FrFT properties for analyzing the Doppler chirp rate of an azimuth-matched filter.

## 3. Proposed Technique

As mentioned in the previous Section, accurate Doppler signal estimation is crucial for generating the final synthetic aperture radar (SAR) image during the azimuth compression process. When the Doppler parameters, specifically the Doppler centroid ($f_{\eta_{c}}$) and chirp rate ($K_{a}$), are mismatched with the expected azimuth matched filter, the final SAR image may be degraded by various unfocused targets. The Doppler centroid can be estimated at the point where the peak of the beam center crosses the target and is largely influenced by Doppler ambiguity. Doppler ambiguity occurs when the Doppler shift extends beyond the azimuth sampling frequency, i.e., outside the Pulse Repetition Frequency (PRF), leading to ghost targets at (*n* ± 1) PRF in the SAR image at *n* PRF. Even though phase-based Doppler compensation techniques [12] are applied, estimating accurate Doppler centroid information can be challenging.

The inaccurate estimation of Doppler chirp centroids results in aliasing effects in the final SAR image, introducing unexpected ghost targets due to the mismatched Doppler centroid frequency. The equation for $S_{2}\left(\tau ,\eta \right)$ with the Doppler centroid error is shown as:

(16) $$S_{2}\left(\tau ,\eta \right)\approx \;A_{0}p_{r}\left[\tau -\frac{2R_{0}}{c}\right]\omega_{a}\left(f_{\eta }-(f_{\eta_{c}}\;\pm \;f_{error})\right)e^{-j\pi f_{0}R_{0}/c}e^{j\pi \frac{f_{\eta }^{2}}{K_{a}}}$$

We can validate the Doppler centroid through mathematical expressions. By processing the Range Doppler Algorithm (RDA), the azimuth-compressed data for extracting the SAR image is denoted in (10). Since the Doppler effect is dominant in the azimuth direction, it remains as a trace in the Doppler centroid form and introduces the Doppler frequency error in $s_{ac}\left(\tau ,\eta \right)$ in the frequency domain. The phase error effect of the Doppler centroid, $e^{f_{error}}$, is defined as:

(17) $$s_{ac}\left(\tau ,\eta \right)=A_{0}p_{r}\left[\tau -\frac{2R_{0}}{c}\right]p_{a}\left(\eta \right)e^{-j\frac{4\pi f_{0}R_{0}}{c}}e^{j(2\pi f_{\eta_{c}}\;\pm \;f_{error})}$$

**Figure 5:**
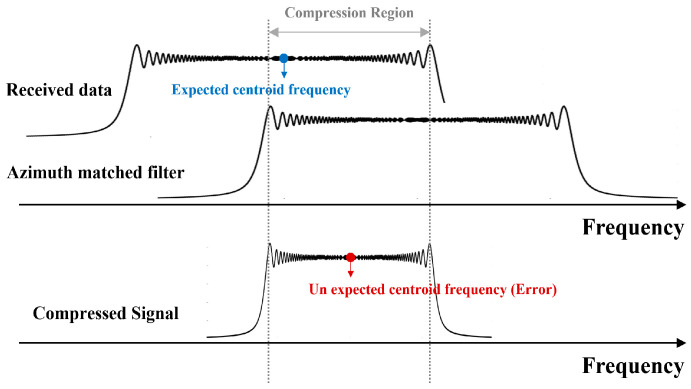
Illustration of mismatching filtering between received data and azimuth-matched filter.

The Doppler centroid frequency remains in the phase of $s_{ac}\left(\tau ,\eta \right)$ in the time domain. Therefore, when the received signals are compressed with an azimuth-matched filter, the center frequency components of the two signals must be the same so that no residual error term remains. That is, if the positions (center frequencies) of the two matching signals are different, the ω-functions (window function) in Equation (8) are multiplied with different locations, and an error term $e^{f_{error}}$ is generated in the matched filtering result, as shown in Figure 5. The Doppler chirp rate, which represents the change in frequency over time, is approximated as a linear change. By (5), the Doppler rate is related to parameters such as velocity and slant range. Since these parameters are variable, it leads to the unexpected rate error by the incorrect Doppler chirp estimation. The Doppler rate with an error term added is defined as follows:

(18) $${K_{a}}^{\prime }\approx \;\frac{2{\left(V_{r}\;\pm \;V_{error}\right)}^{2}}{{\lambda (R}_{0}\;\pm \;R_{error})}$$

A chirp rate error causes inaccurate Doppler estimation, resulting in ghost targets and scattering in the SAR image, which degrade the resolution because the azimuth compression is not executed correctly. The signals before and after azimuth compression are derived in (8) and (10), respectively. By comparing the phase between the $S_{2}\left(\tau ,\eta \right)$ and $S_{ac}\left(\tau ,f_{\eta }\right)$ signals, we can observe the difference in the FrFT domain due to variable $K_{a}$. If the chirp rate of the $S_{2}\left(\tau ,\eta \right)$ signal changes due to unexpected environmental conditions, the azimuth-matched filter can be redefined as $e^{j\pi f_{\eta }^{2}/(K_{a}\;\pm \;K_{a}\prime )}$. This can be denoted as:

(19) $$S_{2}\left(\tau ,\eta \right)\approx \;A_{0}p_{r}\left[\tau -\frac{2R_{0}}{c}\right]\omega_{a}\left(f_{\eta }-f_{\eta_{c}}\right)e^{-\frac{j\pi f_{0}R_{0}}{c}}e^{j\pi f_{\eta }^{2}/(K_{a}\;\pm \;K_{a}\prime )}$$

**Figure 6:**
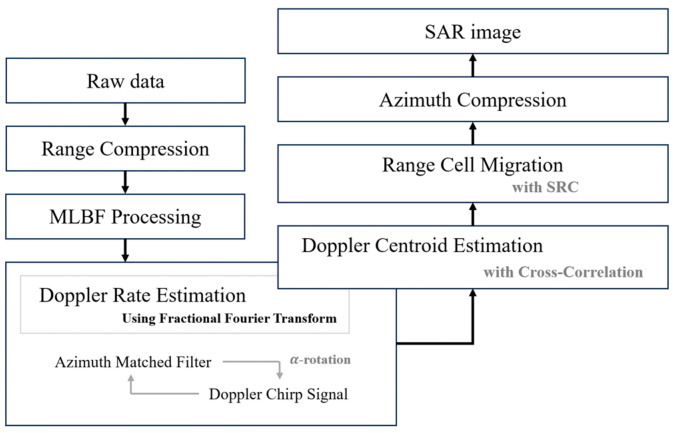
Block scheme of the proposed algorithm.

**Figure 7:**
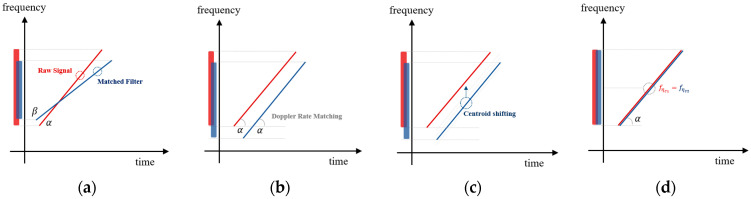
The procedure of processing the proposed technique in the time–frequency domain. (**a**) unexpected raw signal and expected matched filter, (**b**) Doppler rate matching in the Doppler rate estimation, (**c**) Doppler centroid shifting in the Doppler centroid estimation, and (**d**) unexpected raw signal and reconstructed matched filter.

By the mismatched filtering of $K_{a}\prime$, it can remain in the final SAR image. Therefore, in the proposed technique, to accurately extract the Doppler effect from the received data, we estimate the Doppler rate using the FrFT to align the chirp rate of the matched filter with the signal. And then, we estimate the Doppler centroid using cross-correlation to adjust the Doppler centroid of the compensated azimuth-matched filter. The final proposed algorithm is illustrated in Figure 6. After Multi-Look Beat Frequency (MLBF) processing, the proposed algorithm seeks to accurately identify the Doppler chirp pulse signal. The FFT-type MLBF process [22] compensates for the Doppler ambiguity that occurs outside the range of −PRF/2~PRF/2. Our proposed method can accurately estimate the Doppler information (Doppler rate and centroid values) of the received data. Subsequently, the algorithm can shift the corresponding Doppler chirp signal within the range of −PRF/2~PRF/2, applying the ambiguity number estimated by the MLBF process.

(20) $$\sum_{n}f_{\eta_{c}}=\sum_{n}\left(f_{\eta_{c}}^{\prime }+M_{amb}PRF\right)$$

where $f_{\eta_{c}}^{\prime }$ is the Doppler centroid frequency before modifying and $f_{\eta_{c}}$ is the Doppler frequency after modifying. $M_{amb}$ is the ambiguity number estimated by the MLBF process and n is the range count number. Figure 7 provides an example of the unexpected Doppler chirp signal and the azimuth-matched filter, highlighting different chirp rates and Doppler centroids due to the unexpected Doppler effect. The chirp rate can be estimated by the FrFT rotation because both are associated with the rotation angle, indicated by α and β, respectively. One signal shows an increasing frequency rate over time in the time–frequency domain. By FrFT-rotating the two orthogonal axes (time axis and frequency axis), the rotation angle at which the projection value onto the frequency axis is highest can be found. The highest integral value can be considered as the most accurate Doppler chirp rate, and the chirp rate can be calculated using the estimated rotation angle [20]. However, the proposed algorithm not only finds the Doppler chirp rate but also both the “range” and “velocity”, which are derived from the chirp rate at the estimated rotation angle. The chirp rate is associated with the velocity (squared value) and range, which are variable parameters. Specifically, the estimated key parameters of the chirp rate on the FrFT domain are subsequently applied to all other SAR processing stages. This comprehensive approach not only improves the performance of Range Cell Migration Correction (RCMC) and azimuth compression but also enhances the effectiveness of future error compensation algorithms. Thus, the FrFT angle is rotated, based on the two variables, to find the optimal chirp rate that matches the original signal. Using this characteristic, it becomes straightforward to adjust the chirp rate of the azimuth-matched filter by iteratively varying the velocity and range to match the received signal, as shown below:

(21) $$H_{az}\left(f_{\eta }\right)=e^{-j\pi \frac{f_{\eta }^{2}}{K_{a}\;\pm \;∆K_{a}}},\;\;\;\;∆K_{a}=\;\frac{2∆V^{2}}{\lambda ∆R}$$

The velocity of the raw data can be a Doppler parameter estimation for aligning the chirp rate. The error of the platform velocity in the orbit space is caused by unexpected radar motion errors, orbit mismatches, and other factors. Moreover, the error in the range can occur due to the irregular terrain of the target. Based on the chirp rate Equation (9), because the error velocity is more dominant than the error of the range, the parameter of velocity is more weighted in an iterating process for the Doppler rate estimation. The steps of the Doppler rate estimation are introduced as follows:

Step 1. Estimate the chirp rate ($K_{1}$) of the received Doppler signal in the FrFT domain.

Step 2. Estimate the chirp rate ($K_{2}$) of the matched filter in the FrFT domain.

Step 3. Compare each chirp rate value by an iterative process (velocity and range).

Step 4. Stop iterating when $K_{2}$ is equal to $K_{1}$.

After estimating the parameters, the received signal and the matched filter can be aligned to have the same Doppler chirp rate, as illustrated in Figure 7b. Based on the above results, we need to accurately estimate the Doppler centroid frequency, which was previously mentioned as an important component of Doppler information. The Doppler centroid frequencies in each chirp signal are defined as $f_{\eta_{c1}}$ and $f_{\eta_{c2}}$, respectively, and the initial Doppler center frequencies as $f_{\eta_{c1}}$ and $f_{\eta_{c2}}$; the estimates are defined from the Average Cross-Correlation Coefficient (ACCC) estimation method. To align the Doppler centroid, we apply the cross-correlation technique to the proposed estimation method. As shown in Figure 3, the convolution result between ideal chirp pulses appears as a sinc function when perfectly matched. As shown in Figure 7c, to achieve the ideal value of cross-correlation, each signal should be arranged at the same Doppler centroid ($f_{\eta_{c}}$ = $f_{\eta_{c2}}$) based on the chirp rate, which was matched in the previous process. The alignment ratio between each centroid can be inferred from the initial cross-correlation value of the two mismatched signals. There is a method to achieve the highest match by shifting the centroid of the matched filter, and another method to reverse-calculate the centroid value based on the cross-correlation value of the two signals. In the proposed algorithm, the optimized centroid value is estimated using an iterative method. The Doppler centroid of the matched filter is selected for optimization. The Doppler centroid represents the azimuth center frequency of the data, enabling us to use Equation (9) by modifying the Doppler centroid as shown below:

(22) $$H_{az}\left(f_{\eta }\right)=e^{-j\pi \frac{{\left(f_{\eta }\;\pm \;f_{error}\right)}^{2}}{K_{a}}}$$

Through this iterative process of continuously updating the velocity and range parameters, the matched filter becomes an increasingly accurate approximation of the received signal. When the value reaches its maximum, the algorithm terminates the iteration. The procedure for Doppler centroid estimation is shown in Figure 7c,d. The position where the cross-correlation value between the received signal and the estimated signal from FrFT step is maximized can be defined as the Doppler centroid. After completing the overall compensation processing, the raw signal and the azimuth-matched filter are optimized, as shown in Figure 7d.

Figure 7 shows the process of the proposed algorithm to express the details of Figure 6. Figure 7a–c are the procedures of the Doppler rate estimation using the FrFT. The slopes of the received signal and matched filter are different due to the Doppler mismatch in Figure 7a,b. After processing the Doppler rate estimation, the slope of the matched filter is equal to the received signal in Figure 7c. Figure 7c shows the Doppler centroid mismatch between the two signals. After the processing of the Doppler centroid estimation, the matched filter can be aligned with the received signal. Therefore, based on the overall process of our proposed techniques, high-quality SAR images can be generated.

## 4. Experimental Results

**Figure 8:**
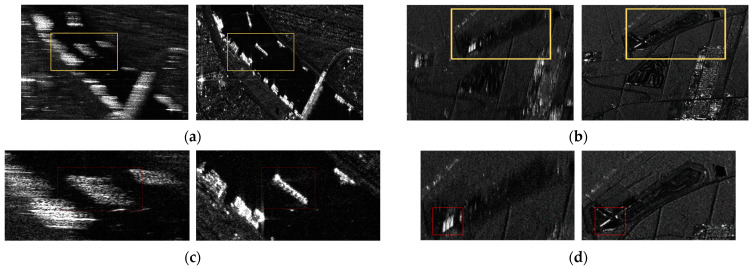
SAR images extracted using the conventional algorithm (**left**) and the proposed algorithm (**right**) from (**a**) raw data I and (**b**) raw data II. The images in (**c**) and (**d**) are the part of the yellow boxes of (**a**,**b**).

To validate the practical performance of the proposed technique, we utilized actual raw data from one of the satellites that are operating in space. The SAR system parameters are listed in Table 1. The satellite operates in the X-band frequency, and although the radar frequency of the three raw datasets is identical, the values of the SAR system parameters such as duration, PRF, and bandwidth vary due to different orbital conditions depending on the monitored regions. We present the comparative processed results between the conventional algorithm and the proposed algorithm, using the same raw data. The SAR image results of the proposed compensation method and the conventional method are given in Figure 8a,b. The difference in performance between the SAR images extracted by the two algorithms is significant. The SAR images produced by the conventional algorithm (left side of Figure 8) are distorted due to incorrect Doppler estimation. In contrast, the SAR images extracted using the proposed algorithm (right side of Figure 8) are clear and accurately focused.

**Figure 9:**
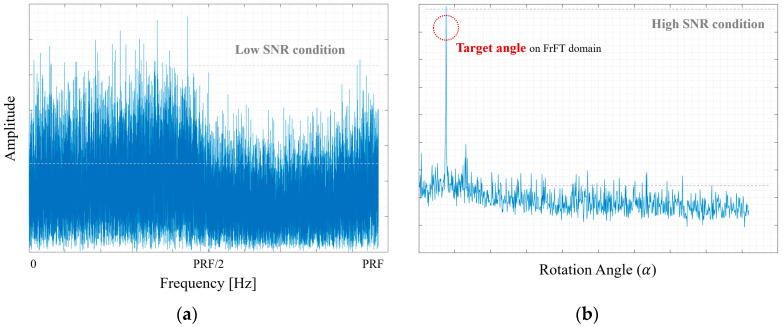
The comparison of the noise environment level between the conventional process and the proposed process: the received signal (**a**) in the FFT domain and (**b**) in the FrFT domain.

When supporting inaccurate Doppler information, the conventional algorithm is significantly out of focus compared to that of the proposed compensation algorithm. Additionally, the expanded images highlighted by the yellow boxes in Figure 8a,b are shown in Figure 8c,d for a more precise comparison. We can find a clear difference in resolution between the two algorithms. From Figure 8c,d, the expanded SAR images, applied to the proposed technique, can be analyzed with various well-focused targets due to the high accuracy of matching between the raw data and matched filter. Thus, even if the SAR system initially supports incorrect Doppler information, the proposed algorithm compensates for this by providing accurate Doppler information, resulting in a clearer and more informative SAR image. Additionally, Table 2 shows the performance of the Doppler estimation. Table 2 shows the Doppler values compensated using the proposed algorithm, derived from the data with existing errors. The proposed algorithm consistently provides accurate Doppler estimation, enhancing the overall SAR image quality. For the analysis of the noise environment level between the conventional process and the proposed process, the power levels of each received signal are compared, as shown in Figure 9. Our proposed method has higher noise resistance than the existing methods. It means that it provides estimates with high accuracy. Table 3 summarizes the advantages of our proposed algorithm, compared with other previous techniques. It shows how well our algorithm is optimized for estimating Doppler signals. These experimental results can validate the effectiveness of our technique in practical applications.

## 5. Conclusions

In this paper, we proposed an advanced algorithm to address the miscalculation of the Doppler chirp rate and Doppler centroid based on the Fractional Fourier Transform (FrFT) and cross-correlation techniques. Through the proposed algorithm, we effectively compensated for the Doppler effects, resulting in the extraction of high-quality synthetic aperture radar (SAR) images. Our results demonstrate a significant performance with simple and streamlined methods, particularly under unexpected environmental conditions. The experimental results using actual satellite raw data validate the practicality and effectiveness of our proposed technique. The SAR images processed with our algorithm exhibited well-focused results and high accuracy compared to those processed with the conventional algorithm. This improvement is evident in the clear boundary lines and forms of targets, which are crucial for accurate terrain analysis and target identification. Our approach offers a robust solution for SAR image processing, adaptable to various platforms such as satellites, aircraft, and automobiles. In particular, SAR via aircraft provides substantial advantages in terrain reconnaissance, while SAR via automobile enhances autonomous driving capabilities. However, the iterative method employed in our algorithm for Doppler centroid estimation, though effective, can be constrained by factors such as the scene size of the raw data and the step size of the iteration. The proposed algorithm is optimized for “velocity and range” errors that may occur in space orbit environments, as it is suitable for satellites. Since sufficient integration in the FrFT domain cannot be achieved in such a nonlinear Doppler slope environment, the error rate can increase. Future research will focus on improving these aspects to obtain more accurate Doppler chirp signal estimates in various environments. We anticipate that the proposed algorithm will contribute to the advancement of SAR techniques across multiple platforms, paving the way for more reliable and accurate remote sensing applications. The continued development and optimization of this algorithm will further enhance its applicability and performance, solidifying its role in the field of synthetic aperture radar.

## Figures and Tables

**Table 1:** Specifications of the SAR system.

System Parameter	Value
Radar Frequency	X-band
Radar Type	Chirp Pulse Radar
Operation Mode	Standard Mode (Side-Looking)

**Table 2:** The values of chirp rate and Doppler centroid processed by the conventional algorithm and the proposed algorithm from each raw data set.

		The Conventional Algorithm	The Proposed Algorithm
Raw data I	Estimated chirp rate	4055.6	4406.6
	Estimated Doppler centroid	−448 Hz	−499 Hz
Raw data II	Estimated chirp rate	4547	4860.5
	Estimated Doppler centroid	815 Hz	765 Hz

**Table 3:** Comparison with various compensation methods.

	Proposed Technique	[14]	[15]	[16]
Process Steps	2 Steps (Iteration)	6 Steps (Iteration)	5 Parameters (Approximated by cases)	Learning Episode (over 1000)
Complexity	Low	Low	High	High
Processing Time	Low (in the orbit)	Medium	Low (in certain cases)	High

## Data Availability

Data are contained within the article.
